# *Staphylococcus aureus*-derived factors induce IL-10, IFN-γ and IL-17A-expressing FOXP3^+^CD161^+^ T-helper cells in a partly monocyte-dependent manner

**DOI:** 10.1038/srep22083

**Published:** 2016-02-26

**Authors:** Sophia Björkander, Lena Hell, Maria A. Johansson, Manuel Mata Forsberg, Gintare Lasaviciute, Stefan Roos, Ulrika Holmlund, Eva Sverremark-Ekström

**Affiliations:** 1 Department of Molecular Biosciences, The Wenner-Gren Institute, Stockholm University, Stockholm, Sweden; 2 Department of Microbiology, Swedish University of Agricultural Sciences, Uppsala, Sweden

## Abstract

*Staphylococcus aureus* (*S. aureus*) is a human pathogen as well as a frequent colonizer of skin and mucosa. This bacterium potently activates conventional T-cells through superantigens and it is suggested to induce T-cell cytokine-production as well as to promote a regulatory phenotype in T-cells in order to avoid clearance. This study aimed to investigate how *S. aureus* impacts the production of regulatory and pro-inflammatory cytokines and the expression of CD161 and HELIOS by peripheral CD4^+^FOXP3^+^ T-cells. Stimulation of PBMC with *S. aureus* 161:2-cell free supernatant (CFS) induced expression of IL-10, IFN-γ and IL-17A in FOXP3^+^ cells. Further, CD161 and HELIOS separated the FOXP3^+^ cells into four distinct populations regarding cytokine-expression. Monocyte-depletion decreased *S. aureus* 161:2-induced activation of FOXP3^+^ cells while pre-stimulation of purified monocytes with *S. aureus* 161:2-CFS and subsequent co-culture with autologous monocyte-depleted PBMC was sufficient to mediate activation of FOXP3^+^ cells. Together, these data show that *S. aureus* potently induces FOXP3^+^ cells and promotes a diverse phenotype with expression of regulatory and pro-inflammatory cytokines connected to increased CD161-expression. This could indicate potent regulation or a contribution of FOXP3^+^ cells to inflammation and repression of immune-suppression upon encounter with *S. aureus*.

Superantigens, like staphylococcal enterotoxins, are the only known virulence factors that deliberately induce activation of the adaptive immune system. They activate large numbers of T-cells via MHC class II-mediated binding to the variable domain of the T-cell receptor (TCR) β-chain^1^, with the exception of staphylococcal enterotoxin H (SEH), which binds to the TCR α-chain^2^. Superantigens suppress immune responses by inducing T-cell exhaustion and anergy^3^. Further, most people have circulating antibodies to superantigens indicating memory formation^4^.

*Staphylococcus aureus* (*S. aureus*) is a human pathogen that frequently causes bacterial infections - but it is also a frequent colonizer on skin and at mucosal surfaces. Certain strains of *S. aureus* produce enterotoxins that can act as superantigens. Today, not much is known regarding the effect of *S. aureus* on peripheral T-helper (T_H_) cell responses, although CD4 T-cells stimulated with pure superantigens express interleukin (IL)-10 and interferon (IFN)-γ^3^,^5^,^6^.

Expression of the transcription factor forkhead box P3 (FOXP3) is characteristic for thymus-derived T-regulatory cells (T_regs_) that develop in the thymus in response to self-antigens, and also for peripherally derived T_regs_ that develop from naive T-cells in the periphery upon activation^7^. Increasing data support that FOXP3^+^ cells show a remarkable functional and phenotypic plasticity. They are able to up-regulate expression of IFN-γ and CXCR3 under T_H_1-polarizing conditions^8^ and also adopt phenotypes equivalent to T_H_2, T_H_17 and T_H_22 effector cells^9^. Recently, it has been shown that FOXP3^+^ cells express the C-type lectin receptor (CLR) CD161, a marker also found on NK-cells and effector/memory T-cells. CD161^+^ T_regs_ more prominently produce IFN-γ and IL-17 upon polyclonal activation compared to the CD161^−^ subpopulation^10^,^11^. Expression of the transcription factor HELIOS is found in most thymus-derived T_regs_ and is therefore suggested to represent T_regs_ of thymic origin^12^. However, the link between HELIOS-expression and thymic origin has been questioned^13^ and lately, HELIOS has been described as an activation marker for both T_reg_ and T_H_-cells^14^,^15^. This indicates that HELIOS^+^ T_regs_ represent a subpopulation of T_regs_ capable of distinct responses rather than identifying thymic origin.

Interestingly, stimulation of peripheral blood mononuclear cells (PBMC) with staphylococcal enterotoxin A (SEA) induces expression of FOXP3 and an increased expression of cytotoxic T-lymphocyte-associated protein 4 (CTLA-4) indicating a conversion of naive or effector T-cells into T_regs_^6^. Further, up-regulation of FOXP3-expression occurs in CD25^−^ CD4 T-cells upon TCR-stimulation^16^,^17^,^18^,^19^, however this up-regulation was found not to be exclusive for cells with regulatory/suppressive capabilities^17^,^18^,^19^.

Here, we investigated how soluble products from several *S. aureus*-strains (cell free supernatant, CFS) influence human CD4 T-cells, and particularly CD4^+^FOXP3^+^ cells, in terms of CD161 and HELIOS-expression and induction of IL-10, IFN-γ and IL-17A. We demonstrate that the enterotoxin-gene-expressing *S. aureus* 161:2-strain potently increases the percentage of FOXP3^+^ cells among the CD4^+^ T-cell population and induces a diverse phenotype in FOXP3^+^ cells with production of regulatory and pro-inflammatory cytokines connected to increased expression of CD161, in a partly monocyte-dependent manner. Together, these data increase the knowledge of how *S. aureus* impacts CD4^+^FOXP3^+^ cells in the early stages of infection.

## Results

### *S. aureus* 161:2-CFS stimulates CD4 T-cells to express of IL-10, IFN-γ and IL-17A

**Figure 1:**
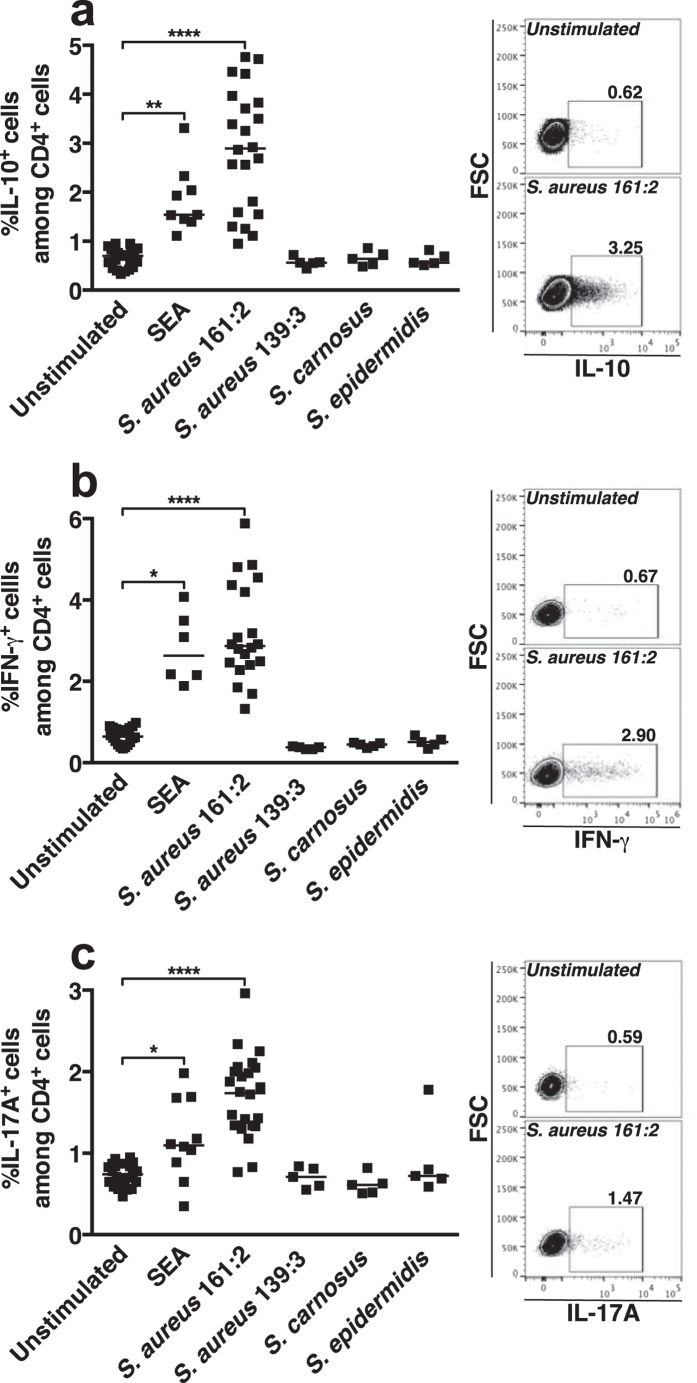
*S. aureus* 161:2-CFS stimulates CD4 T-cells to express of IL-10, IFN-γ and IL-17A. **(a–c)** The percentages and representative facs-plots of CD4 T-cells expressing IL-10 (**a**), IFN-γ (**b**) and IL-17A (**c**) after 24-hour stimulation with SEA (n = 6–10), *S. aureus* 161:2-CFS (n = 16–19), *S. aureus* 139:3-CFS (n = 5), *S. carnosus* TM300-CFS (n = 5) and *S. epidermidis* KX293A1-CFS (n = 5). The horizontal line represents the median within each group.

To confirm T_H_-responsiveness towards *S. aureus* 161:2, we stimulated PBMC for 24 hours with SEA or with CFS derived from *S. aureus* 161:2, *S. aureus* 139:3 (a strain that has previously been described not to induce T-cell activation^20^) or from two non-pathogenic staphylococci: *S. carnosus* TM300 or *S. epidermidis* KX293A1, isolated from food and skin respectively. The PBMC were then analysed by flow cytometry and live T_H_-cells were gated based on the expression of CD4 and the Live/Dead-marker. Stimulation with *S. aureus*-161:2-CFS or with SEA induced expression of the pro-inflammatory cytokines IFN-γ and IL-17A but also of the regulatory cytokine IL-10. In contrast, CFS from *S. aureus* 139:3, *S. carnosus* TM300 or *S. epidermidis* KX293A1 did not induce CD4 T-cell cytokine-production (Fig. 1a–c).

### *S. aureus* 161:2-CFS induces FOXP3-expression in CD4 T-cells

**Figure 2:**
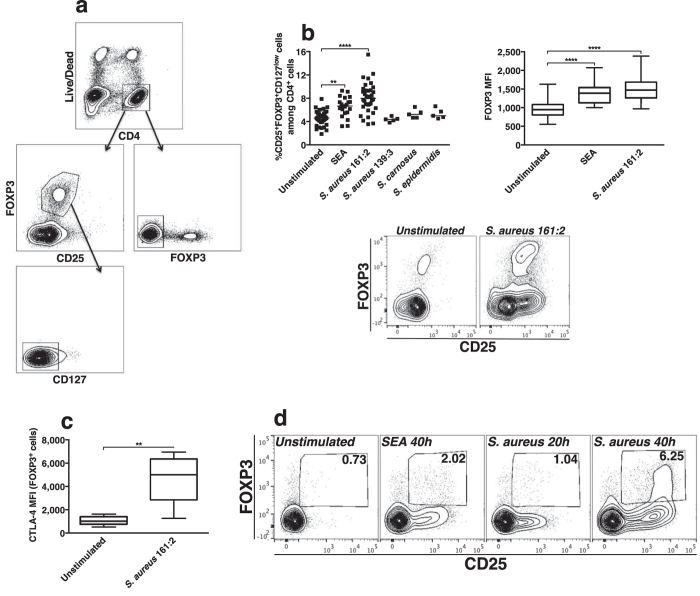
*S. aureus* 161:2-CFS induces FOXP3-expression in CD4 T-cells. Lymphocytes were gated according to FSC and SSC properties. The further gating strategy is displayed in **(a)**: Live CD4^+^ T-cells were gated either as CD25^+^FOXP3^+^ and CD127^low/neg^ (left panel) or as FOXP3^−^ (right panel). These two populations are referred to as FOXP3^+^ and FOXP3^−^ cells respectively in the text and figures. **(b)** The percentage of CD25^+^FOXP3^+^CD127^low^ cells among CD4^+^ T-cells in PBMC-cultures after 24-hour stimulation with SEA (n = 21), *S. aureus* 161:2-CFS (n = 36), *S. aureus* 139:3-CFS (n = 5), *S. carnosus* TM300-CFS (n = 5) or *S. epidermidis* KX293A1-CFS (n = 5) (2b, top, left) and mean fluorescence intensity (MFI) of FOXP3-expression (2b, top, right). The dot plot shows one representative staining of CD25 vs FOXP3-expression (2b, bottom). **(c)** CTLA-4-expression in FOXP3^+^ cells after 24-hour stimulation with *S. aureus* 161:2-CFS (n = 8). **(d)** Representative stainings of CD25 vs FOXP3-expression in purified CD4^+^CD25-depleted T-cell-cultures either unstimulated or after stimulation with SEA or *S. aureus* 161:2-CFS. Boxes cover data values between the 25^th^ and 75^th^ percentiles, with the central line as median. For scatter dot plots, the horizontal lines represent the median within each group.

Bacterial superantigens are known to induce a regulatory phenotype in CD4^+^CD25^−^ T-cells in terms of up-regulation of FOXP3-expression and IL-10-production^6^. We therefore stimulated PBMC with the different staphylococcal-CFS and analysed for the expression of CD25, FOXP3 and CD127 in/on live CD4^+^ T-cells with flow cytometry. In the analysis, CD4^+^ T-cells were divided into having a CD25^+^FOXP3^+^CD127^low^ T_reg_-like phenotype or as being FOXP3^−^ cells (Fig. 2a). We observed a significant increase in the percentage of FOXP3^+^ cells and in FOXP3-expression after 24-hour stimulation with *S. aureus* 161:2-CFS but not after stimulation with the other staphylococci that do not produce enterotoxins (Fig. 2b). Also, intracellular CTLA-4-expression in FOXP3^+^ cells increased upon stimulation (Fig. 2c). To investigate whether *S. aureus*-CFS could induce FOXP3-expression in T_H_-cells, we isolated CD4^+^CD25-depleted T-cells by magnetic separation and stimulated these cells with SEA and *S. aureus* 161:2-CFS in the presence of autologous monocytes. Indeed, both stimuli induced *de novo* expression of CD25 and FOXP3 in comparison with unstimulated conditions (Fig. 2d).

### *S. aureus* 161:2-CFS induces expression of regulatory- and pro-inflammatory cytokines in FOXP3^+^ CD4 T-cells

**Figure 3:**
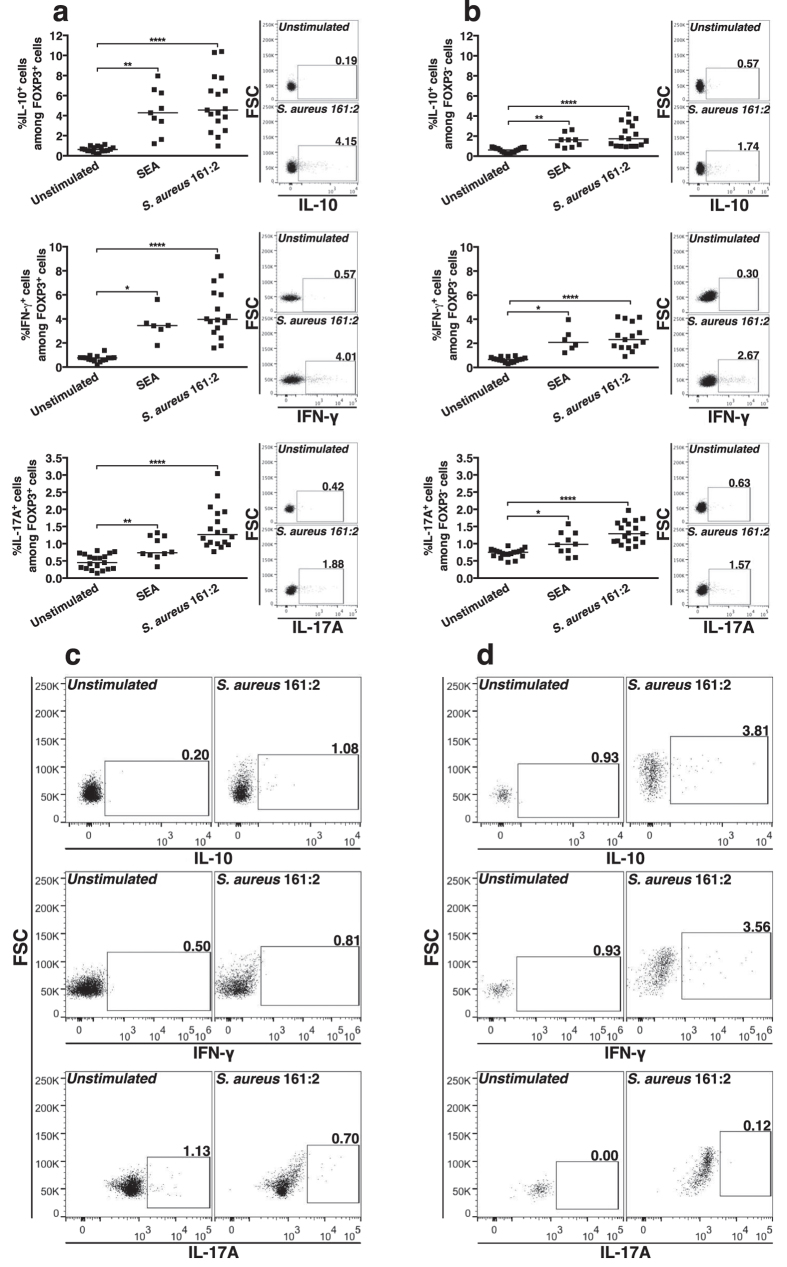
*S. aureus* 161:2-CFS induces expression of regulatory- and pro-inflammatory cytokines in FOXP3^+^ CD4 T-cells. (**a)** The percentages of FOXP3^+^ cells and **(b)** the percentages of FOXP3^−^ cells that express IL-10 (upper panel), IFN-γ (middle panel) or IL-17A (lower panel) after 24-hour stimulation with SEA (n = 6–10) or *S. aureus* 161:2-CFS (n = 16–18). (**c**) Representative facs-plots of cytokine-expression in CD25^+^FOXP3^+^ cells in isolated CD4^+^CD25^high^ T-cell-cultures after 40-hour stimulation with *S. aureus* 161:2-CFS. (**d**) Representative facs-plots of cytokine-expression in cells with *de novo* expression of CD25 and FOXP3 in CD4^+^CD25-depleted T-cell-cultures after 40-hour stimulation with *S. aureus* 161:2-CFS. For scatter dot plots, the horizontal lines represent the median within each group.

Next we investigated the capacity of FOXP3^+^ cells to express cytokines in response to *S. aureus* 161:2**-CFS. PBMC were stimulated with *S. aureus* 161:2-CFS or SEA and analysed by flow cytometry. FOXP3^+^ and FOXP3^−^ cells were gated as described above (Fig. 2a) and the percentages of cytokine-expressing cells within these both populations were determined. FOXP3^+^ cells primarily expressed IL-10 and IFN-γ, and to a lesser extent IL-17A; this was also the case for FOXP3^−^ cells (Fig. 3a,b). We then isolated both CD4^+^CD25-depleted T-cells and CD4^+^CD25^high^ T-cells from PBMC-cultures by magnetic separation and stimulated them in the presence of autologous monocytes. Cells with *de novo* co-expression of CD25 and FOXP3 induced in stimulated cultures of CD4^+^CD25-depleted T-cells expressed IL-10 and IFN-γ while cells with co-expression of CD25 and FOXP3 in stimulated cultures of CD4^+^CD25^high^ T-cell-cultures remained unresponsive (Fig. 3c,d).

### *S. aureus* 161:2-CFS induces IL-10-expressing cells within the CD161^+^ subpopulation of FOXP3^+^ CD4 T-cells

**Figure 4:**
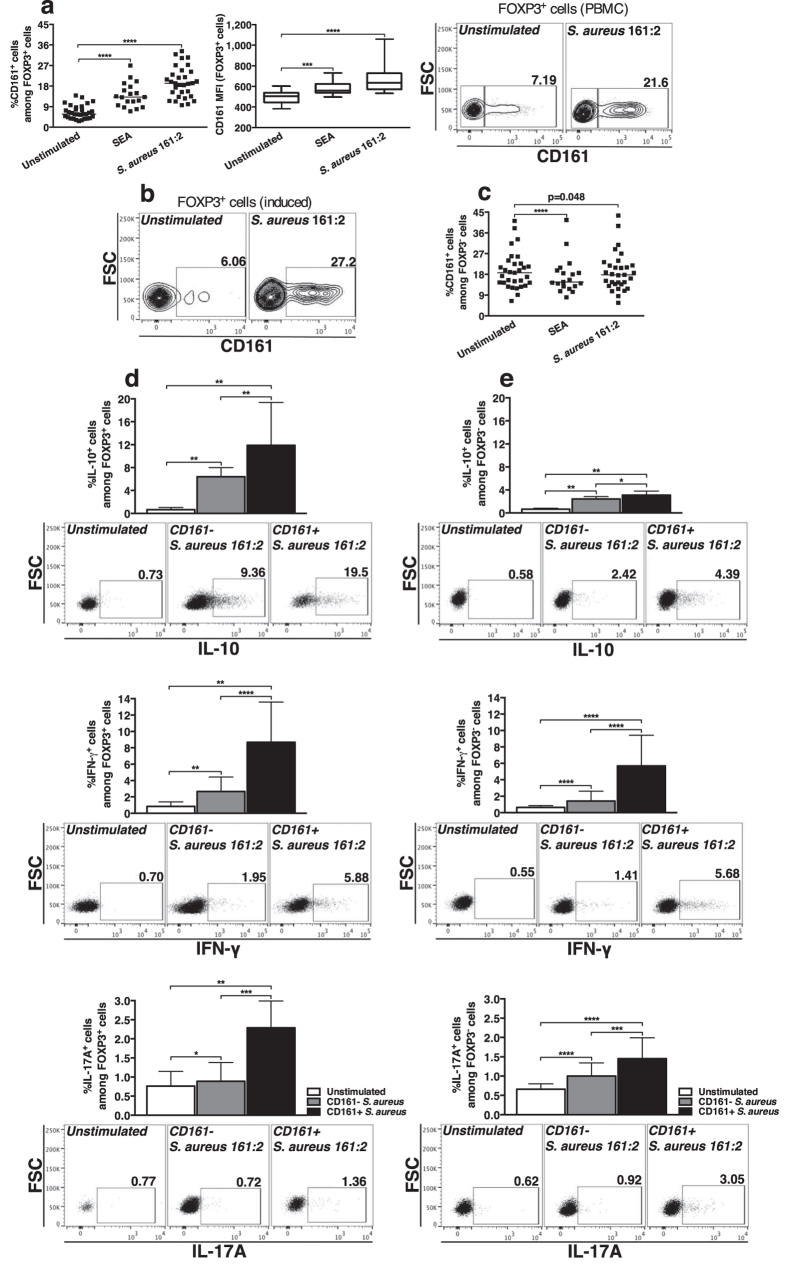
*S. aureus* 161:2-CFS induces IL-10-expressing cells within the CD161^+^ subpopulation of FOXP3^+^ CD4 T-cells. (**a)** The percentage of CD161^+^ cells within the FOXP3^+^ population and CD161-expression on FOXP3^+^ cells in PBMC-cultures after 24-hour stimulation with SEA (n = 19) or *S. aureus* 161:2-CFS (n = 31). Facs-plot shows one representative staining of CD161-expression on FOXP3^+^ cells in PBMC-cultures. **(b)** Representative facs-plot of CD161-expression on CD25^+^FOXP3^+^ cells in stimulated CD4^+^CD25-depleted T-cell-cultures. **(c)** The percentage of CD161^+^ cells within the FOXP3^−^ population as described in (**a**). **(d**,**e)** The percentages of IL-10^+^, IFN-γ^+^ and IL-17A^+^ cells within the CD161^−^ subpopulation (grey bars) or the CD161^+^ subpopulation (black bars) both for FOXP3^+^ cells (**d**) and FOXP3^−^ cells (**e**) after 24-hour stimulation with *S. aureus* 161:2-CFS (n = 8–19). For scatter dot plots, the horizontal line represents the median within each group. Boxes cover data values between the 25^th^ and 75^th^ percentiles, with the central line as median. Bars show medians with interquartile range.

CD161 is suggested to identify T-cells, including FOXP3^+^ cells, with a high capacity to produce pro-inflammatory cytokines like IL-17A and IFN-γ^10^,^11^. We therefore stimulated PBMC as described above and investigated the expression of CD161 by FOXP3^+^ CD4 T-cells. *S. aureus* 161:2-CFS and SEA had a profound impact on the percentage of CD161^+^ cells within the FOXP3^+^ population as well as on CD161 surface expression (Fig. 4a). Further, CD161-expression was induced on cells with *de novo* expression of CD25 and FOXP3 in stimulated CD4^+^CD25-depleted T-cell cultures (Fig. 4b). In contrast, the percentage of FOXP3^−^CD161^+^ cells was slightly decreased in PBMC-cultures after stimulation with *S. aureus* 161:2-CFS or SEA (Fig. 4c). In stimulated PBMC-cultures, there was a higher percentage of IFN-γ^+^ and IL-17A^+^ cells within the CD161^+^ subpopulation, both for FOXP3^+^ and FOXP3^−^ cells. Interestingly, there was also a higher percentage of IL-10^+^ cells within the CD161^+^ subpopulation compared to the CD161^−^ counterpart (Fig. 4d,e). The same pattern could be observed after stimulation with SEA (Supplementary Fig. S1). *S. aureus*-stimulation did not alter the percentage of CD161^+^ cells within the total CD4 T-cell population or induce CD161-expression in isolated, naive CD4 T-cells cultured with autologous monocytes (Supplementary Fig. S2)

### Expression of CD161 and HELIOS separates FOXP3^+^ cells into four distinct populations with differences in cytokine-expression

**Figure 5:**
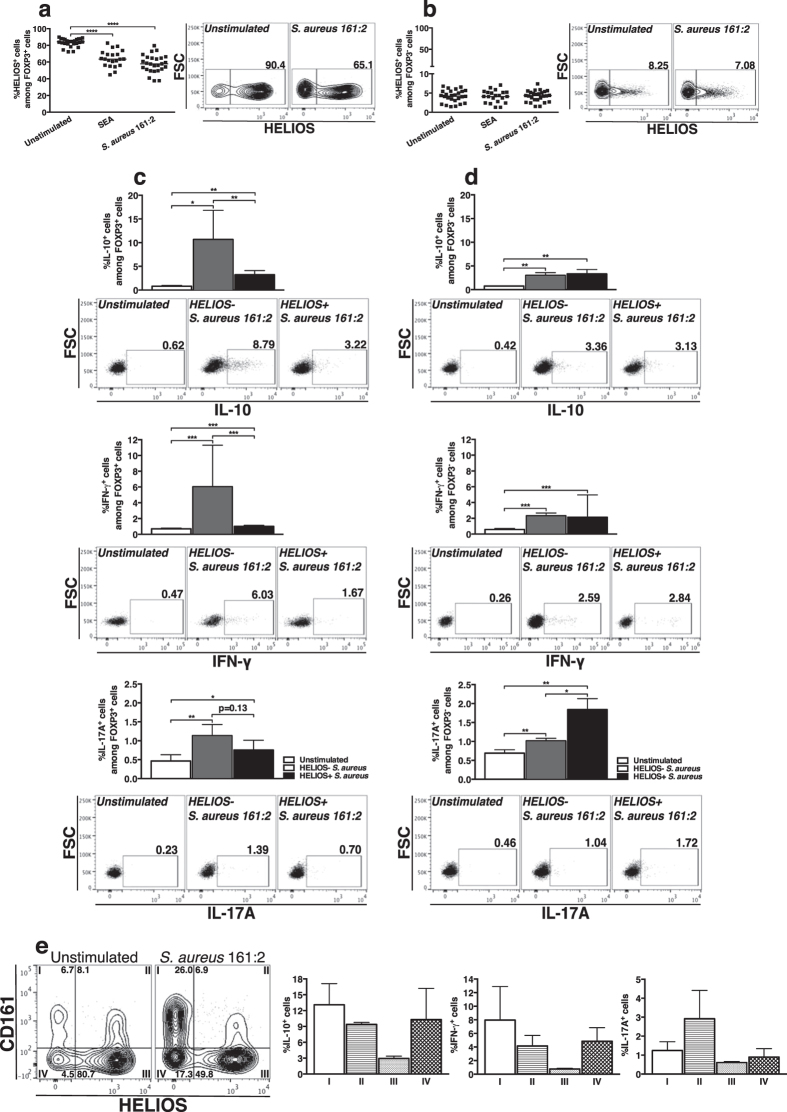
Expression of CD161 and HELIOS separates FOXP3^+^ cells into four distinct populations with differences in cytokine-expression. (**a,b**) The percentages of HELIOS^+^ cells within the FOXP3^+^ (**a**) or FOXP3^−^ (**b**) populations after 24-hour stimulation with SEA (n = 21) or *S. aureus* 161:2-CFS (n = 26). (**c,d**) The percentages of IL-10^+^, IFN-γ^+^ and IL-17A^+^ cells within the HELIOS^−^ subpopulation (grey bars) or the HELIOS^+^ subpopulation (black bars) both for FOXP3^+^ cells (**c**) and FOXP3^−^ cells (**d**) after 24-hour stimulation with *S. aureus* 161:2-CFS (n = 7–11). (**e**) Representative facs-plot showing the division of FOXP3^+^ cells into four populations (I–IV) based on the expression of CD161 and HELIOS and the percentages of IL-10^+^, IFN-γ^+^ and IL-17A^+^ cells within populations I-IV after 24-hour stimulation with *S. aureus* 161:2-CFS (n = 9–11). For scatter dot plots, the horizontal line represents the median within each group. Bars show medians with interquartile range.

To further characterize the cytokine-expressing FOXP3^+^ cells, we investigated HELIOS-expression after stimulation of PBMC with *S. aureus*-CFS. The percentage of HELIOS^+^ cells within the FOXP3^+^ population decreased after stimulation with *S. aureus* 161:2-CFS (Fig. 5a), while the percentage of FOXP3^−^ cells expressing HELIOS remained unaltered (Fig. 5b). There were significantly higher percentages of IL-10^+^ and IFN-γ^+^ cells, but not of IL-17A^+^ cells, within the HELIOS^−^ subpopulation compared to the HELIOS^+^ subpopulation of FOXP3^+^ cells (Fig. 5c), a pattern that was not observed in the FOXP3^−^ subpopulation (Fig. 5d). As CD161-expression is linked to production of pro-inflammatory cytokines while HELIOS-expression is connected to lack of cytokine-production by T_regs_ we investigated the co-expression of these markers on FOXP3^+^ cells. In unstimulated conditions, we observed a similar percentage of CD161^+^HELIOS^+^ and CD161^+^HELIOS^−^ cells within the FOXP3^+^ population. After stimulation with *S. aureus* 161:2-CFS, the percentage of CD161^+^HELIOS^−^ cells was increased (Fig. 5e, left). We then divided the FOXP3^+^ cells into four populations: I: CD161^+^HELIOS^−^, II: CD161^+^HELIOS^+^, III: CD161^−^HELIOS^+^ and IV: CD161^−^HELIOS^−^ cells. In general, population III (CD161^−^HELIOS^+^) was the least responsive population. A similar percentage of IL-10^+^ and IFN-γ^+^ cells were found within the other three populations, while another pattern was observed for IL-17A^+^ cells, where the highest percentage of positive cells was seen in population II (CD161^+^HELIOS^+^) (Fig. 5e, right). SEA-stimulation induced similar results (data not shown) and overall, FOXP3^+^ cells were similarly activated by CD3/CD28-beads as by *S. aureus* 161:2-CFS (Supplementary Fig. S3).

### Monocytes are involved in *S. aureus*-mediated activation of FOXP3^+^ cells

**Figure 6:**
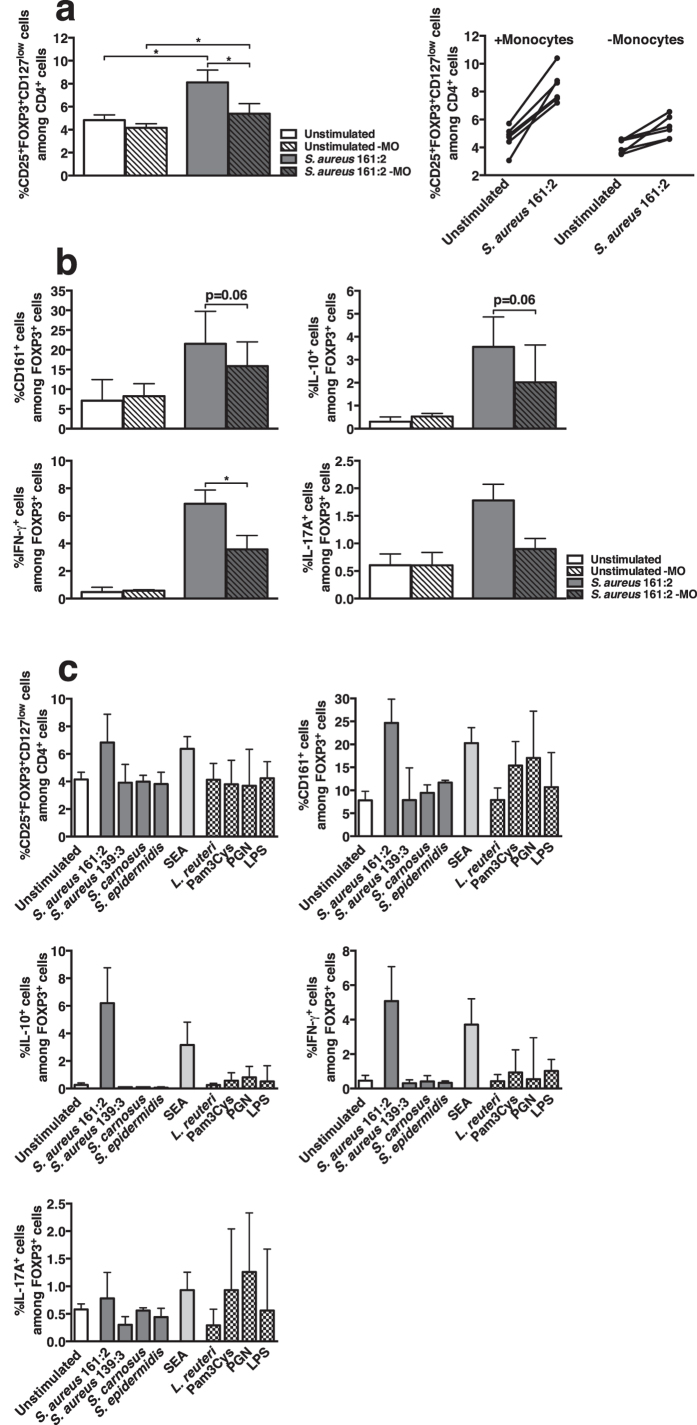
Monocytes are involved in the induction of FOXP3-expression and in the functional responses of FOXP3^+^ cells. (**a)** Left: the percentage of FOXP3^+^ cells in whole PBMC-cultures (open bars) or in monocyte-depleted PBMC-cultures (-MO) (striped bars) either unstimulated (white bars) or after 24-hour stimulation with *S. aureus* 161:2-CFS (grey bars) (n = 6). Right: the percentage of FOXP3^+^ cells shown for individual donors in whole PBMC (+monocytes) or in monocyte-depleted PBMC (-monocytes). **(b)** The percentage of CD161^+^, IL-10^+^, IFN-γ^+^ and IL-17A^+^ cells within the FOXP3^+^ population as described in (**a**) (n = 5–6). **(c)** The percentage of FOXP3^+^ cells and the percentages of FOXP3^+^ cells that express CD161, IL-10, IFN-γ or IL-17A in monocyte-depleted PBMC-cultures after 20 hours of co-culture with purified monocytes that had been pre-stimulated for four hours with *S. aureus* 161:2-CFS, *S. aureus* 139:3-CFS, *S. carnosus* TM300-CFS, *S. epidermidis* KX293A1-CFS, SEA, *L. reuteri* DSM 17938-CFS, Pam3Cys, PGN or LPS (n = 3–6). Bars show medians with interquartile range.

Superantigens activate T-cells in a non-specific manner through bridging of MHC class II molecules on antigen presenting cells (APC) with the TCR. We therefore analysed the potential role for monocytes in *S. aureus*-CFS-induced activation of FOXP3^+^ cells. Depletion of CD14^+^ cells from PBMC-cultures clearly, but not completely, diminished the effect of *S. aureus* 161:2 on FOXP3^+^ cells (Fig. 6a,b). To further investigate the role of APC, we purified monocytes and stimulated them with CFS from the different *S. aureus* strains and *Lactobacillus (L) reuteri* DSM 17938 or with SEA, Pam3Cys, peptidoglycan (PGN) or lipopolysaccharide (LPS). Purified monocytes produced IL-6 in culture supernatants following incubation with all stimuli except SEA and expression of both HLA-DR and CD86 was up-regulated after stimulation with *S. aureus* 161:2-CFS and SEA (Supplementary Fig. S4). Pre-stimulation of monocytes with *S. aureus* 161:2-CFS or with SEA was sufficient to induce an increased percentage of FOXP3^+^ cells as well as an increased percentage of FOXP3^+^CD161^+^ upon subsequent co-culture with autologous monocyte-depleted PBMC (Fig. 6c). IL-10 and IFN-γ-expressing FOXP3^+^ cells could mainly be detected after stimulation with *S. aureus* 161:2-CFS and SEA. In addition, there was an increase in IL-17A-expressing FOXP3^+^ cells after stimulation with *S. aureus* 161:2-CFS and SEA, however we also observed a similar or higher percentage after stimulation with Pam3Cys or PGN (Fig. 6c). Depletion of monocytes from PBMC-cultures affected the percentage of IL-10 and IL-17A-expressing FOXP3^−^ cells and pre-stimulated monocytes induced cytokine-expression in FOXP3^−^ cells (Supplementary Fig. S5).

## Discussion

In this study, we show that stimulation with *S. aureus* 161:2-CFS profoundly activates the FOXP3^+^ CD4 T-cell compartment in a partly monocyte-dependent manner by induction of CD161^+^FOXP3^+^ cells with a diverse phenotype characterized by expression of IL-10 as well as IFN-γ and IL-17A. Further we show that when co-expressed with CD161, HELIOS-expressing FOXP3^+^ CD4 T-cells were able to produce both IL-10 and IFN-γ.

We observed an increased expression of FOXP3 in CD4 T-cells in PBMC-cultures upon stimulation with *S. aureus* 161:2-CFS, but not with the other staphylococci strains tested. Further, *S. aureus* 161:2-CFS induced *de novo* expression of FOXP3 in CD4^+^CD25-depleted T-cell cultures. These results are in line with studies by others, where Taylor *et al*. also showed an increase in CD25^+^FOXP3^+^ cells after stimulation with pure superantigen; while isolated T_regs_ did not proliferate, up-regulation of FOXP3 by the CD4^+^CD25^−^ population was observed^6^. On the other hand, Rabe *et al*. reported that neonatal non-T_regs_ are converted into functionally suppressive CD25^+^CD127^low^ cells after exposure to UV-killed, enterotoxin-producing *S. aureus* in the presence of pre-existing autologous T_regs_ and monocytes^21^. Together with these previous studies, our findings strongly suggest that *S. aureus* induces an up-regulation of FOXP3-expression by FOXP3^−^ CD4 T-cells.

We could also clearly demonstrate that *S. aureus*-derived soluble components potently induced a diverse response in CD4^+^CD25^+^FOXP3^+^CD127^low^ cells, but also in FOXP3^−^ cells, in PBMC-cultures. Notably, both the FOXP3^+^ and the FOXP3^−^ populations produced the immune regulatory cytokine IL-10 after stimulation with *S. aureus* 161:2-CFS or SEA. Interestingly, and in agreement with our findings, *S. aureus* has been suggested to deliberately induce IL-10-production through superantigenic stimulation as a way of escaping the immune system and prolonging tolerance^6^,^22^. The role for IL-10 in the function of FOXP3^+^ cells, including T_regs_, is not clear and the consequences of IL-10-production by circulating FOXP3^+^ cells upon stimulation with *S. aureus*-CFS seen in our study might therefore be multiple and indicative of an immunosuppressive and/or regulatory phenotype, however this needs to be investigated further.

In addition to IL-10, we show that FOXP3^+^ cells are capable of expressing IFN-γ and IL-17A after stimulation with *S. aureus* 161:2-CFS. It is suggested that T_regs_ mediate suppression against specific T_H_-subsets by adopting their transcriptional program^23^,^24^,^25^. FOXP3^+^ cells expressing IFN-γ and IL-17A may therefore rapidly develop upon *S. aureus*-induced activation to suppress initial immune responses. In contrast, production of pro-inflammatory cytokines normally associated with effector T-cells could act to polarize surrounding cells and repress immune-suppression in the early stages of infection. Interestingly, expansion of endogenous T_regs_ by using IL-2C or adoptive transfer of *ex vivo* expanded T_regs_ in HLA-DR3 transgenic mice, which potently respond to staphylococcal enterotoxin B (SEB) and mimic human toxic shock syndrome (TSS), did not mitigate TSS. Further, systemic levels of IFN-γ and IL-10 were even more elevated in transgenic mice treated with SEB+IL-2C compared to mice treated with only SEB^26^, showing that expanding FOXP3^+^ cells could be an important source of cytokines upon superantigen-exposure. Whether *S. aureus* promotes induction of cytokine-expressing FOXP3^+^ cells from effector T-cells, or an adoption of pro-inflammatory properties in pre-existing T_regs_ remain an open question. However, our data indicate that the cytokine-expressing FOXP3^+^ cells are mainly induced from the CD4^+^FOXP3^−^ population as cells with *de novo* expression of CD25 and FOXP3 expressed cytokines following stimulation. In contrast, FOXP3^+^ cells in cultures of isolated CD4^+^CD25^high^ T-cells did not expand or express cytokines upon stimulation. The cytokine-responses of FOXP3^+^ cells upon stimulation with *S. aureus* 161:2-CFS show that these cells adopt a diverse phenotype upon microbial challenge and can change depending on the type of immune activation.

Recently, expression of the CLR CD161 was described to identify T-cells with a common transcriptional and functional phenotype^27^, and the production of IFN-γ and IL-17 by FOXP3^+^ cells is linked to CD161-expression^10^,^11^. We observed an increase in the percentage of CD161^+^ cells among FOXP3^+^ cells after stimulation with *S. aureus* 161:2-CFS. Also, we found a higher percentage of IFN-γ^+^ and IL-17A^+^ cells within the CD161^+^ subpopulation of FOXP3^+^ cells. Still, a significant percentage of CD161^−^ cells produced IFN-γ and IL-17A indicating that the capacity to produce pro-inflammatory cytokines is not exclusive for the CD161^+^ subpopulation of FOXP3^+^ cells. More surprisingly, there was a higher percentage of IL-10^+^ cells within the CD161^+^ subpopulation, showing that CD161-expression by FOXP3^+^ cells associates with a more general cytokine-expressing phenotype, possibly reflecting an increased need for cytokine-expressing FOXP3^+^ cells after *S. aureus*-encounter. Since the percentage of CD161^+^ cells within the whole CD4 T-cell population did not alter with stimulation, this would suggest a conversion of FOXP3^−^CD161^+^ cells into FOXP3^+^CD161^+^ cells rather than an expansion of CD161^+^ cells. This up-regulated FOXP3^+^CD161^+^ subpopulation could have functional differences compared to the small basal population of FOXP3^+^CD161^+^ cells. Further, *S. aureus* 161:2-CFS did not induce CD161-expression in naive CD4 T-cells.

The use of HELIOS to separate thymus-derived and peripherally-derived T_regs_ has been frequently debated during the last years^13^,^15^,^28^ as HELIOS^−^ cells can be found within the population of naive (CD45RA^+^) FOXP3^+^ T_regs_. However, HELIOS^−^ T_regs_ are reported to be more potent cytokine-producers compared to HELIOS^+^ T_regs_^13^,^29^,^30^, but to have a similar suppressive capacity^13^. In the present study, the majority of FOXP3^+^ cells expressed HELIOS at unstimulated conditions. Stimulation with *S. aureus* 161:2-CFS or SEA reduced the percentage of HELIOS^+^ cells within the FOXP3^+^ population indicating up-regulation of FOXP3-expression by the HELIOS^−^ T_H_-population, while the percentage of FOXP3^−^ cells expressing HELIOS was low and did not alter with stimulation. Importantly, HELIOS^−^ but also HELIOS^+^ cells expressed cytokines after stimulation with *S. aureus* 161:2-CFS, indicating that the HELIOS^+^ subpopulation might be more pleiotropic than previously described. Even though we observed a higher percentage of IL-17A^+^ cells within the HELIOS^+^ subpopulation of FOXP3^−^ CD4 T-cells, neither IL-10 nor IFN-γ correlated with HELIOS-expression for FOXP3^−^ cells.

We further divided the FOXP3^+^ cells into four populations based on the expression of both CD161 and HELIOS, as cytokine-expression within the FOXP3^+^ population was differentially correlated with these markers. After stimulation with *S. aureus* 161:2-CFS, the percentage of CD161^+^HELIOS^−^ cells increased, which was not observed for CD161^+^HELIOS^+^ cells. However, the CD161^+^HELIOS^−^ and CD161^+^HELIOS^+^ subpopulations both potently expressed IL-10 and IFN-γ after *S. aureus* 161:2-stimulation showing that these cytokines is more tightly linked to expression of CD161 than to lack of HELIOS. Interestingly, the highest percentage of IL-17A^+^ cells was found within the CD161^+^HELIOS^+^ subpopulation, strengthening the observation that IL-17A-production by FOXP3^+^ cells is not linked to a lack of HELIOS. For all three cytokines, CD161^−^HELIOS^+^ cells were close to unresponsive. This suggests that CD161-expression should be considered when investigating functional responses of HELIOS-expressing FOXP3^+^ cells.

Superantigens activate T-cells in a polyclonal fashion through bridging of MHC class II molecules on APC with the TCR. Indeed, the presence of monocytes was important, however not indispensible, for *S. aureus* 161:2-CFS-induced FOXP3-expression or for the activation of FOXP3^+^ cells. These results suggest that other cells expressing MHC class II contribute to T-cell activation by superantigens. It should be noted that we have not identified the main activating component(s) in the *S. aureus* 161:2-CFS. However, pure SEA induced a similar activation of the FOXP3^+^ CD4 T-cells in comparison to *S. aureus* 161:2-CFS, indicating that the main stimulatory component in the CFS is enterotoxin, although not directly measured. Also, a 4h stimulation of purified monocytes with the 161:2-strain or SEA was enough to mediate activation of autologous FOXP3^+^ cells after subsequent co-culture, while CFS from *S. aureus* 139:3, *S. carnosus* or *S. epidermidis* did not induce monocyte-mediated activation of FOXP3^+^ cells. LPS-primed monocytes have been shown to enhance production of pro-inflammatory cytokines by T_regs_ in the presence of T-cell stimuli^31^. In our study, monocytes stimulated with Pam3Cys or PGN induced activation of FOXP3^+^ cells upon co-culture, with increased percentages of CD161^+^ and IL-17A^+^ cells, without the addition of further T-cell stimulation. As IL-6 is important for IL-17A-production^32^, monocyte-secreted IL-6 following Toll-like receptor-stimulation might activate FOXP3^+^ cells to produce IL-17A suggesting that *S. aureus* can mediate activation also via pattern recognition receptor-ligands.

Taken together, our data suggest that *S. aureus* 161:2-CFS promotes monocyte-mediated induction of FOXP3^+^ cells, with diverse responses indicating either powerful regulation or a possible contribution of FOXP3^+^ cells as effector cells during early stages of *S. aureus*-encounter. The increased FOXP3^+^ population contained a higher percentage of CD161^+^ cells and a lower percentage of HELIOS^+^ cells suggesting conversion of effector CD161^+^HELIOS^−^ T_H_-cells into FOXP3^+^ cells. Expression of IL-10, IFN-γ and IL-17A in FOXP3^+^ cells correlated with both CD161- and HELIOS-expression. To our knowledge, we are the first to show that IL-10-expression in FOXP3^+^ cells is connected to CD161-expression. This study provides new insights into the functional phenotypes of FOXP3^+^ cells responding to *S. aureus* and widens the knowledge of the function of CD161 and HELIOS in FOXP3^+^ CD4 T-cells.

## Materials and Methods

### Subjects and Ethics Statement

A total of 38 healthy adult volunteers (18 women and 20 men) were included in this study, which was approved by the Regional Ethic’s Committee at the Karolinska Institute, Stockholm, Sweden [Dnr 04-106/1 and 2014/2052-32]. All study subjects gave their informed written consent. All samples were coded and stored as stated in the approved ethical application and the methods were carried out in accordance with the approved guidelines. It is not possible to connect published data to any individual.

### Bacterial cell free supernatants

Cell free supernatants (CFS) from *S. aureu*s 161:2 (expressing genes for SEA and SEH) and *S. aureus* 139:3 (both kind gifts from Åsa Rosengren, The National Food Agency, Uppsala, Sweden), non-pathogenic *S. carnosus* TM300 isolated from food and *S. epidermidis* KX293A1 (own isolate, unpublished) was used for this study. The staphylococci were cultured in BHI broth (Merck, Darmstadt, Germany) at 37 °C for 72 hours in still culture. For co-culture experiments, CFS from *L. reuteri* DSM 17938 (kind gift from Biogaia AB, Stockholm, Sweden) cultured in MRS broth (Oxoid, Hampshire, UK) at 37 °C for 20 hours in still culture was used. All bacterial supernatants were separated from the pellet by centrifugation at 3400g. Supernatants were then sterile-filtered (0.2 μm) and frozen at −20 °C until used. For stimulation of PBMC, a final concentration of 2.5% supernatant was used. Extensive testing was performed to determine the optimal concentration of bacterial supernatant that could maximally stimulate lymphocytes in PBMC-cultures without affecting cell viability.

### Peripheral blood mononuclear cell isolation

Venous blood was collected in heparinized vacutainer tubes (BD Biosciences Pharmingen, San Jose, CA, USA) and diluted with RPMI-1640 supplemented with 20 mM HEPES (HyClone Laboratories, Inc, South Logan, UT, USA). The PBMC were isolated by Ficoll-Hypaque (GE Healthcare Bio-Sciences AB, Uppsala, Sweden) gradient separation. The PBMC were used directly or washed in RPMI-1640 and diluted in freezing medium containing 40% RPMI-1640, 50% FCS (Gibco by Invitrogen, Carlsbad, CA, USA) and 10% dimethyl sulphoxide (Sigma Aldrich, St Louis, MO, USA), gradually frozen in a freezing container (Mr Frosty, Nalgene Cryo 1 °C; Nalge Co, Rochester, NY, USA) and stored in liquid nitrogen until analysed.

### *In vitro* activation of PBMC

PBMC were thawed and washed before counting and exclusion of non-viable cells by trypan blue staining. Cells were re-suspended to a final concentration of 0.5–2 × 10^6^ cells/ml in cell culture medium (RPMI-1640 supplemented with 20 mM HEPES, penicillin (100 U/ml), streptomycin (100 μg/ml), L-glutamine (2 mM) (all from HyClone Laboratories Inc) and 10% heat-inactivated fetal calf serum (Gibco by Life Technologies). The cells were added to flat-bottomed 96-well plates (Costar, Cambridge, UK) and incubated for 24 hours at 37 °C in 6% CO_2_ atmosphere with cell culture medium alone as negative control, with 2.5% of *S. aureus*-CFS or with 20 ng/ml of SEA (Sigma Aldrich). In addition, the Dynabeads Human T-Activator CD3/CD28-beads (Gibco by Life Technologies) were used at 2:1 (cell:bead) ratio as positive control. GolgiStop/Monensin (IL-10, IFN-γ) or GolgiPlug/Brefeldin A (IL-17A) (both BD Biosciences) was added during the last four hours of incubation to prevent extracellular transport of produced cytokines. Prior to the main experiments, suitable concentrations and kinetics were determined.

### Monocyte purification or depletion and co-culture of purified monocytes with autologous monocyte-depleted PBMC

The human CD14 positive selection kit (StemCell Technologies, Grenoble, France) was used to purify monocytes or to deplete monocytes from PBMC-cultures according to the instructions of the manufacturer. Monocyte-depleted PBMC were stimulated with 2.5% *S. aureus*, 20 ng/ml of SEA or CD3/CD28-beads at 2:1 (cell:bead) ratio for 24 hours with GolgiStop/Monensin present during the last four hours of incubation and analysed with total PBMC serving as control. Alternatively, 5 × 10^4^ purified monocytes were incubated for four hours with 2.5% *S. aureus*-CFS, 20 ng/ml of SEA, 2.5% *L. reuteri* DSM 17938-CFS or with 100 ng/ml of ultrapure LPS, 10 μg/ml of Peptidoglycan (PGN) or 10 μg/ml of Pam3Cys (all from Invivogen, San Diego, CA, USA) in a v-shaped 96-well plate. After incubation, the supernatants were removed by centrifugation and the monocytes were washed extensively with cell culture media before 4 × 10^5^ autologous monocyte-depleted PBMC were added and cells were co-cultured for 20 hours with GolgiStop/Monensin present during the last four hours of incubation. For investigation of monocyte activation, purified CD14^+^ cells were stimulated with 2.5% *S. aureus*-CFS, 20 ng/ml of SEA, 2.5% *L. reuteri* DSM 17938-CFS, 100 ng/ml of LPS, 10 μg/ml of PGN or 10 μg/ml of Pam3Cys during four or 16 hours, stained with CD14 FITC (clone: B159), CD86 APC (clone: 2331) and HLA-DR PE (clone: G46-6) (all from BD Biosciences) and analysed by flow cytometry. Purity was assed for all donors by staining with CD3 PE-Cy7 (clone: SK7) (Biolegend) and CD14 FITC (clone: B159) (BD Biosciences). The mean percentage of CD14^+^ cells was 0,49 in the monocyte-depleted PBMC and 97,4 after purification.

### Isolation of naive CD4 T-cells, CD4^+^CD25^high^ T-cells and CD4^+^CD25-depleted T-cells

The human naive CD4^+^ T-cell enrichment kit (Stemcell Technologies) was used to negatively select for CD4^+^CD45RO^−^ cells from PBMC according to the instructions from the manufacturer. The average percentage of live CD4^+^ T-cells was 94% and the average percentage of CD45RA^+^CD45RO^−^ cells among live CD4^+^ T-cells was 99.2% (four donors). The human CD4^+^CD25^high^ T-cell isolation kit (Stemcell Technologies) was used to positively select for CD4^+^CD25^high^ cells (average percentage of CD25^+^ cells among live CD4^+^ T-cells for 2 donors: 55.6%) or to deplete CD25^+^ cells from the CD4 T-cell population (average percentage of CD25^+^ cells among live CD4^+^ T-cells for 2 donors: 1.64%) according to the instructions from the manufacturer. The isolated, naive CD4 T-cells were stimulated with 2.5% *S. aureus* 161:2-CFS for 48 h in the presence of autologous monocytes at a 12:1 T-cell-monocyte ratio. The isolated CD4^+^CD25^high^ or the CD4^+^CD25-depleted cells were stimulated with 2.5% *S. aureus* 161:2-CFS for 20h or 40h in the presence of autologous monocyte at a 1:1 T-cell-monocyte ratio. Cells were cultured in the presence of 50 ng/ml of human recombinant IL-2 (Peprotech, Rocky Hill, NJ, USA).

### Flow Cytometry

After incubation, cells were harvested and transferred to v-shaped staining plates and washed twice in cold PBS. The cells were stained with the LIVE/DEAD Fixable Dead Cell Stain Kit-Aqua (Invitrogen by Life Technologies) for 15 min at RT and thereafter washed with PBS. Afterwards, cells were incubated with 10% human serum in FACS-wash buffer (PBS, 2mM EDTA and 0.1% BSA) for 10 minutes to block Fc-receptors and then stained with titrated amounts of the following cell surface antibodies: CD4 FITC (clone: RPA-T4), CD25 APC-H7 (clone: M-A251), CD127 PE-Cy7 (clone: HIL-7R-M21) (all from BD Biosciences) or CD161 PerCP-Cy5.5 (clone: HP-3G10), CD161 BV421 (clone: HP-3G10) (both from Biolegend, San Diego, CA, USA). After staining, cells were washed with FACS-wash buffer. For intracellular staining, the Transcription factor buffer set (BD Biosciences) was used according to the instructions from the manufacturer. After extracellular staining, cells were fixed, permeabilised and stained with titrated amounts of the following intracellular antibodies: FOXP3 PE (clone: 2590/C7), CD152/CTLA-4 BV421 (clone: BNI3), IFN-γ PerCP-Cy5.5 (clone: B27), IFN-γ APC (clone: B27), IL-17A V450 (clone: N49-653) (all from BD Biosciences) or IL-17A PE-Cy7 (clone: BL168), IL-10 APC (clone: JES3-9D7), IL-10 BV421 (clone: JES3-9D7), HELIOS APC (clone: 22F6) (all from Biolegend). Stained cells were washed in FACS-wash buffer and analysed within two hours by using the FACSVerse instrument and the FACSSuite software (both BD Biosciences). For most experiments, data was acquired from flow cytometry analyses of whole PBMC. Then, lymphocytes were gated based on forward and side scatter properties. The Live/Dead marker was used to further gate for the live CD4^+^ T-cell population, which was analysed as a whole or divided into CD4^+^FOXP3^−^ cells or CD4^+^CD25^+^FOXP3^+^CD127^low/neg^ cells. The percentage of CD127^low/neg^ cells within the CD25^+^FOXP3^+^ gate was >90%. These two populations are referred to as FOXP3^−^ and FOXP3^+^ cells in the text and figures. For some experiments, data was acquired from flow cytometry analyses of pre-isolated CD4^+^CD25^high^ or pre-isolated CD4^+^CD25-depleted T-cells. In these cases, the expression of CD25 and FOXP3 and cytokines was investigated on the isolated cells after stimulation. Corresponding isotype-matched antibodies or unstimulated cells were used as negative controls. The results were based either on the percentage of positive cells or on the mean surface expression of receptors per cell defined as geometrical mean fluorescence intensity (MFI). Analysis was done with FlowJo Software (TreeStar, Ashland, OR, USA).

### Interleukin-6 ELISA

IL-6 levels (pg/ml) in cell culture supernatants from purified monocytes stimulated as described above was determined by using sandwich ELISA (MabTech AB, Nacka, Sweden) according to the instructions from the manufacturer. The optical density was determined using a micro-plate reader (Molecular Devices Corp, Sunnyvale, CA) set at 405 nm. Results were analysed using SoftMax Pro 5.2 rev C (Molecular Devices Corp).

### Statistics

The GraphPad Prism 6 software (GraphPad Software, La Jolla, CA, USA) was used for data presentation and statistical analysis. Data were considered non-parametrical and results are displayed as medians with or without interquartile range. The non-parametrical Friedman Test was used for multiple comparisons. Comparison of two parameters within the same individual was made with the Wilcoxon matched pairs test. No outlier or extreme values were excluded from any statistical analysis. The differences were considered significant if p < 0.05. *p < 0.05, **p ≤ 0.01, ***p ≤ 0.001, ****p ≤ 0.0001.

## Additional Information

**How to cite this article**: Björkander, S. *et al*. *Staphylococcus aureus*-derived factors induce IL-10, IFN-γ and IL-17A-expressing FOXP3^+^CD161^+^ T-helper cells in a partly monocyte-dependent manner. *Sci. Rep*. **6**, 22083; doi: 10.1038/srep22083 (2016).

## Supplementary Material

Supplementary Information
