# Supplementary material for: Staphylococcus aureus-derived factors induce IL-10, IFN-γ and IL-17A-expressing FOXP3+CD161+ T-helper cells in a partly monocyte-dependent manner
Source: Sci Rep. 2016 Feb 26;6:22083. doi: 10.1038/srep22083 (PMC4768154; doi:10.1038/srep22083)
Supplement: Supplementary Information [file srep22083-s1.pdf]

Staphylococcus aureus-derived factors induce IL-10, IFN-γ and IL-17A-expressing FOXP3+CD161+ T-helper cells in a partly monocyte-dependent manner

Sophia Björkander[1], Lena Hell[#1], Maria A Johansson[#1], Manuel Mata Forsberg[#1], Gintare Lasaviciute[1], Stefan Roos[2], Ulrika Holmlund[1], Eva Sverremark-Ekström[*1]

[1]Department of Molecular Biosciences, The Wenner-Gren Institute, Stockholm University, Stockholm, Sweden

[2]Department of Microbiology, Swedish University of Agricultural Sciences, Uppsala, Sweden

[#]LH, MAJ and MMF contributed equally to this work

[*]**Corresponding author:**

Eva Sverremark-Ekström

Department of Molecular Biosciences, The Wenner-Gren Institute, Stockholm University, Stockholm Sweden

Phone: +46 8 164178, e-mail: eva.sverremark@su.se

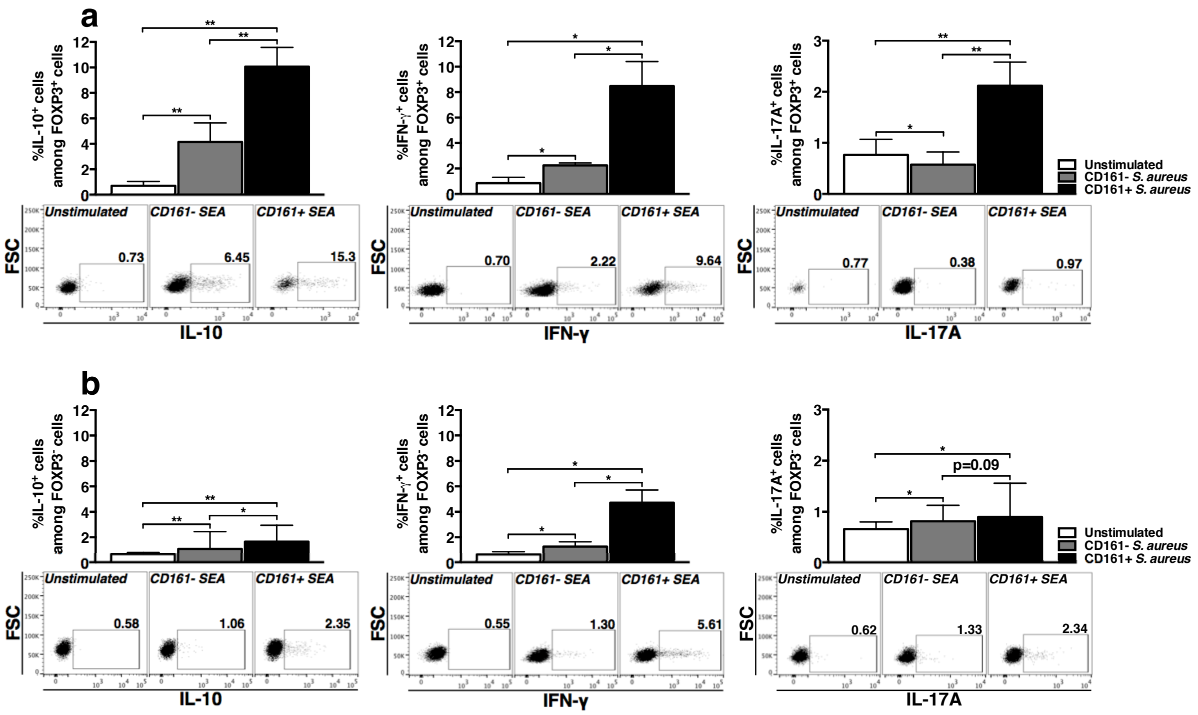

Supplementary Figure S1. SEA induces IL-10-expressing cells within the CD161$^+$ subpopulation of FOXP3$^+$ CD4 T-cells. The percentage of IL-10$^+$, IFN-γ$^+$ and IL-17A$^+$ cells within the CD161$^-$ subpopulation (grey bars) or the CD161$^+$ subpopulation (black bars) both for FOXP3$^+$ cells **(a)** and FOXP3$^-$ cells **(b)** after 24-hour stimulation of PBMC with SEA (n=6-10). Bars show medians with interquartile range.

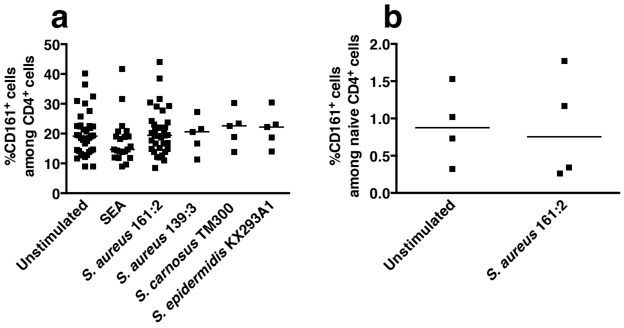

Supplementary Figure S2. **Staphylococcal-CFS does not alter the percentage of CD161$^+$ cells within the CD4 T-cell population. (a)** The percentage of CD161$^+$ cells within the CD4 T-cell population after 24-hour stimulation of PBMC with SEA, *S. aureus* 161:2-CFS, *S. aureus* 139:3-CFS, *S. carnosus* TM300-CFS or *S. epidermidis* KX293A1-CFS (n=5-36). **(b)** The percentage of CD161$^+$ cells within purified naive CD4 T-cells after 48-hour stimulation with *S. aureus* 161:2-CFS (n=4). The horizontal line represents the median within each group.

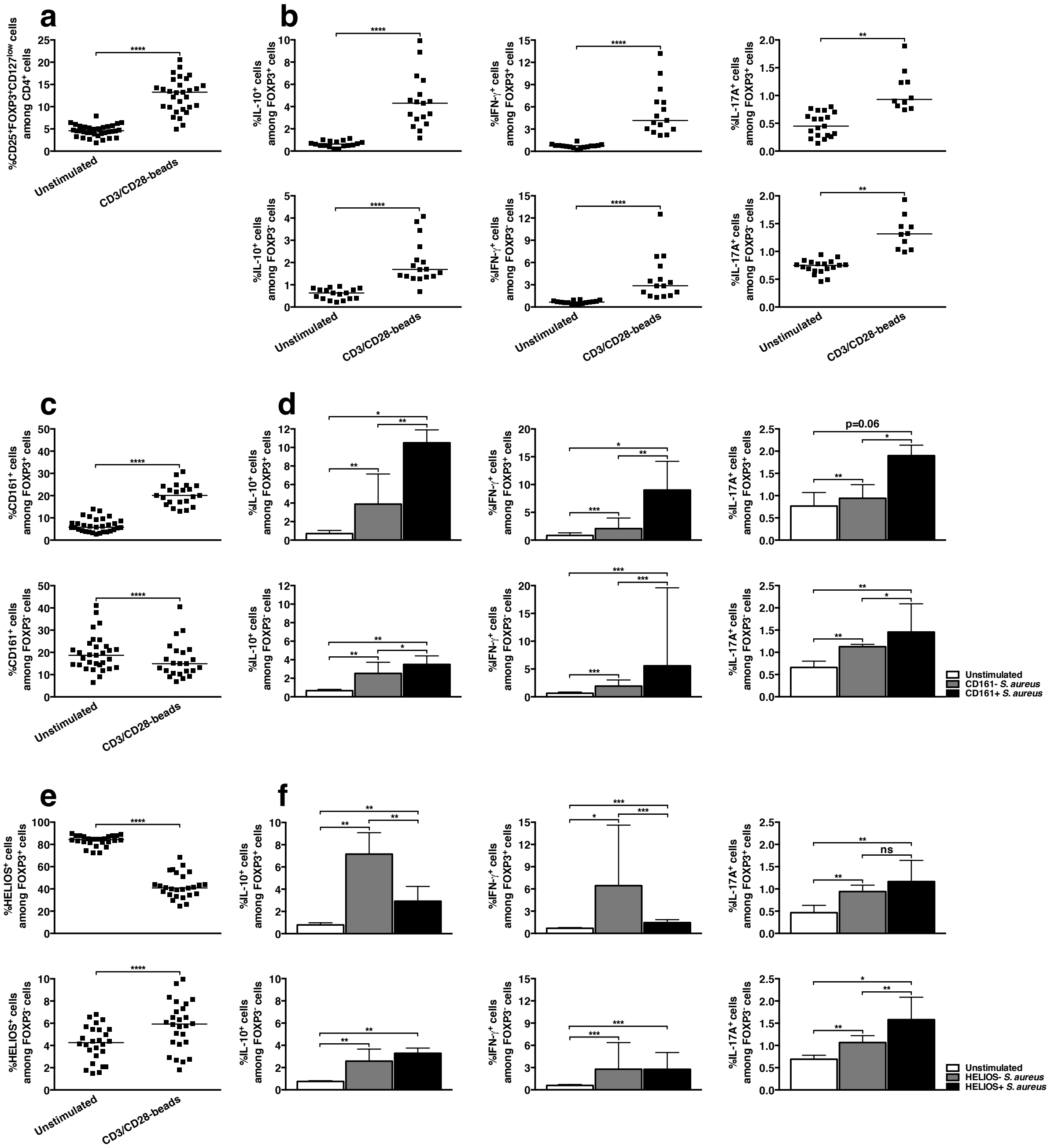

Supplementary Figure S3. FOXP3$^+$ CD4 T-cells respond similarly towards CD3/CD28-beads and to *S. aureus* 161:2-CFS. PBMC were stimulated with CD3/CD28-beads for 24 hours and then analysed by flow cytometry. **(a)** The percentage of CD25$^+$FOXP3$^+$CD127^{low} cells within the CD4$^+$ T-cell population (n=30). **(b)** The percentages of IL-10$^+$ (left), IFN-γ$^+$ (middle) and IL-17A$^+$ (right) cells within the FOXP3$^+$ (upper panel) and FOXP3$^-$ (lower panel) populations (n=10-17). **(c)** The percentages of CD161$^+$ cells within the FOXP3$^+$ and FOXP3$^-$ populations (n=22-23). **(d)** The percentage of IL-10$^+$ (left), IFN-γ$^+$ (middle) and IL-

17A$^+$ (right) cells within the CD161$^-$ subpopulation (grey bars) or the CD161$^+$ subpopulation (black bars) of both FOXP3$^+$ cells (upper panel) and FOXP3$^-$ cells (lower panel) (n=9-11). **(e)** The percentages of HELIOS$^+$ cells within the FOXP3$^+$ and FOXP3$^-$ populations (n=26). **(f)** The percentage of IL-10$^+$ (left), IFN-γ$^+$ (middle) and IL-17A$^+$ (right) cells within the HELIOS$^-$ subpopulation (grey bars) or the HELIOS$^+$ subpopulation (black bars) of both FOXP3$^+$ cells (upper panel) and FOXP3$^-$ cells (lower panel) (n=9-11). For scatter dot plots, the horizontal line represents the median within each group. Bars show medians with interquartile range.

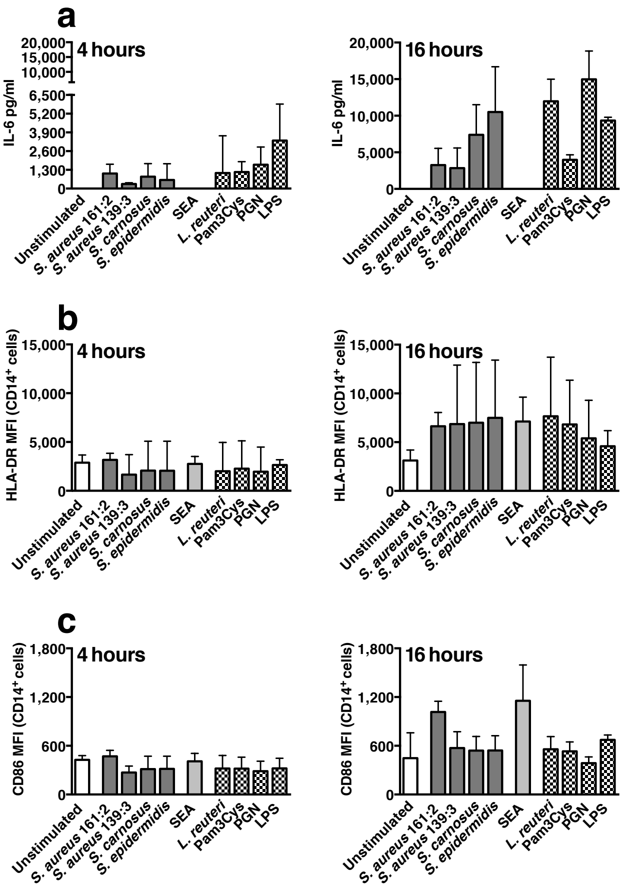

Supplementary Figure S4. Staphylococci-CFS-induced activation of purified monocytes. **(a)** Levels of secreted IL-6 (pg/ml) in culture supernatants from purified monocytes stimulated for four or 16 hours with *S. aureus* 161:2-CFS, *S. aureus* 139:3-CFS, *S. carnosus* TM300-CFS, *S. epidermidis* KX293A1-CFS, SEA, *L. reuteri* DSM 17938, Pam3Cys, PGN or LPS. Values below detection limit were set to 0.01 (n=3-8). **(b-c)** Cell surface expression measured as MFI of HLA-DR (b) and CD86 (c) on purified monocytes stimulated as described in (a) (n=3-8). Bars show medians with interquartile range.

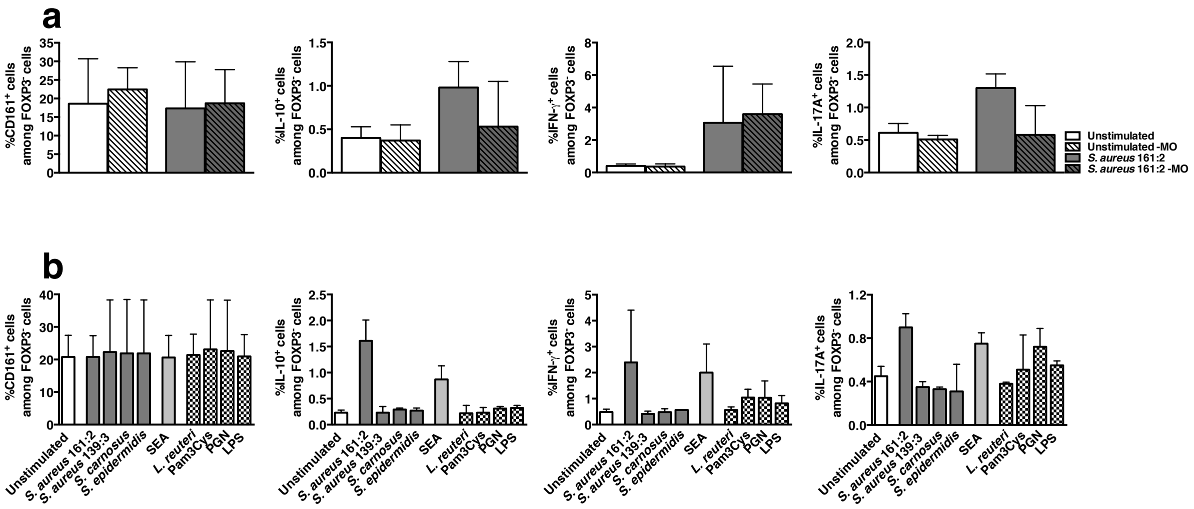

Supplementary Figure S5. Monocytes are partly involved in *S. aureus* 161:2-CFS-induced activation of CD4^{+}FOXP3^{-} cells. (a) The percentages of CD161^{+}, IL-10^{+}, IFN-γ^{+} or IL-17A^{+} cells within the FOXP3^{-} population of CD4 T-cells in whole PBMC-cultures (open bars) or in monocyte-depleted PBMC-cultures (-MO) (striped bars) either unstimulated (white bars) or after 24-hour stimulation with *S. aureus* 161:2-CFS (grey bars) (n=5-6). **(b)** The percentages of FOXP3^{-} cells expressing CD161, IL-10, IFN-γ or IL-17A in monocyte-depleted PBMC-cultures after 20 hours of co-culture with purified monocytes that had been pre-stimulated for four hours with *S. aureus* 161:2-CFS, *S. aureus* 139:3-CFS, *S. carnosus* TM300-CFS, *S. epidermidis* KX293A1-CFS, SEA, *L. reuteri* DSM 17938-CFS, Pam3Cys, PGN or LPS (n=3-6). Bars show medians with interquartile range.
